# Treatment of multiple adjacent gingival recessions using leucocyte- and platelet-rich fibrin with coronally advanced flap: a 12-month split-mouth controlled randomized clinical trial

**DOI:** 10.1007/s00784-024-05694-3

**Published:** 2024-05-01

**Authors:** Atacan Yavuz, Hatice Selin Güngörmek, Leyla Kuru, Başak Doğan

**Affiliations:** 1 Department of Periodontology, Institute of Health Sciences, Marmara University, Istanbul, Türkiye; 2 Department of Periodontology, Faculty of Dentistry, Marmara University, Istanbul, 34854 Türkiye; 3 Department of Oral Health Science and Translational Research, College of Dental Medicine, Nova Southeastern University, Fort Lauderdale, Florida USA

**Keywords:** Connective tissue, Gingival recession, Plastic surgery, Platelet-rich fibrin, Surgical flaps

## Abstract

**Objective:**

This split-mouth randomized study aimed to assess efficacy of leucocyte-platelet-rich fibrin (L-PRF) versus connective tissue graft (CTG) in achieving root coverage (RC) for multiple adjacent gingival recessions (MAGRs) throughout 12-month period.

**Materials and methods:**

The study enrolled 59 teeth from 12 patients with Miller Class I MAGRs ≥ 2 mm on bilateral or contralateral sides. Patients were randomly assigned to receive coronally advanced flap (CAF) with either CTG (control) or L-PRF (test) treatment. Various parameters, including plaque and gingival index, clinical attachment level, recession depth, probing depth, recession width (RW), papilla width (PW), keratinized tissue width (KTW), gingival thickness (GT), percentage of RC, complete root coverage (CRC), and location of the relative gingival margin concerning the cemento-enamel junctions (GMCEJ) after CAF, were recorded at baseline, 3-, 6-, and 12-months post-surgery. On June 29, 2021 the study was registred to ClinicalTrials.gov (NCT04942821).

**Results:**

Except KTW and GT gain, all clinical parameters, RC, and CRC were similar between the groups at all follow-up periods (*p* > 0.05). The higher GT and KTW gains were detected in the control group compared to test group at 12 months (*p* < 0.05). Both RC and CRC were positively associated with initial PW and GMCEJ, but negatively with initial RW (*p* < 0.05).

**Conclusions:**

The current study concludes that L-PRF were equally effective as CTG in treating MAGRs in terms of RC and CRC. Additionally, RC and CRC outcomes appeared to be influenced by GMCEJ, PW, and RW.

**Clinical relevance:**

L-PRF could represent a feasible substitute for CTG in treating MAGRs.

## Introduction

Gingival recession (GR) requires accurate treatment due to aesthetic concerns, root caries, dentinal hypersensitivity or cervical abrasion, and challenges in maintaining plaque control [1, 2]. Several surgical procedures have been suggested for treating single GR achieving various degree of success in terms of complete root coverage (CRC) [3]. In a recent meta-analysis, as a result of its predictable results, the gold standard procedure for achieving root coverage (RC) is still the combination of connective tissue graft (CTG) and coronally advanced flap (CAF) [2]. Addressing multiple adjacent gingival recessions (MAGRs) presents a formidable challenge for clinicians striving for CRC. This complexity arises from dealing with a larger surgical field characterized by substantial anatomic variability, encompassing factors such as a wide avascular recipient bed, prominent root, shallow vestibule depth, and variations in recession depths (RD) and keratinized tissue width (KTW) [4, 5]. The use of CTG in MAGR treatment is limited by its size and thickness, and the procedure is associated with drawbacks such as second-site morbidity, post-operative bleeding, and patient discomfort [6]. Seeking alternatives to mitigate these issues, clinicians have explored various biomaterials like enamel matrix derivatives, acellular dermal matrix, or collagen matrix in combination with CAF. However, the clinical outcomes of these biomaterials do not match those of CTG [1]. In recent decades, platelet-rich concentrations have revolutionized healing and regeneration in dentistry [7]. Platelets release growth factors such as vascular endothelial and platelet-derived growth factor, adhesion factors, and cytokines [8, 9].

Leukocytes, integral components in platelet concentrates, contribute to immune regulation and anti-infectious properties [10]. Leukocyte and platelet-rich fibrin (L-PRF), second-generation platelet concentrate, is produced without the use of gelling agents or anticoagulants [11]. L-PRF comprises a three-dimensional tetra-molecular fibrin consisting of stem cells, platelets, and cytokines. The clot’s initial portion, adjacent to the red cell base, holds the highest platelet concentration and regenerative potential [12]. Growth factors crucial for cell proliferation and migration, microvascularization development, and guiding epithelial cell migration can be sustainedly released due to the L-PRF membrane [13]. While L-PRF has been evaluated for RC in single [14–18] and multiple [19–23] GR treatments, there are limited clinical split-mouth design studies assessing its effectiveness in MAGR treatment with 12-month follow-up [20]. Thus, this study aims to compare the RC effectiveness of CAF + L-PRF with CAF + CTG in MAGR treatment after a 12-month period.

## Materials and methods

**Fig. 1:**
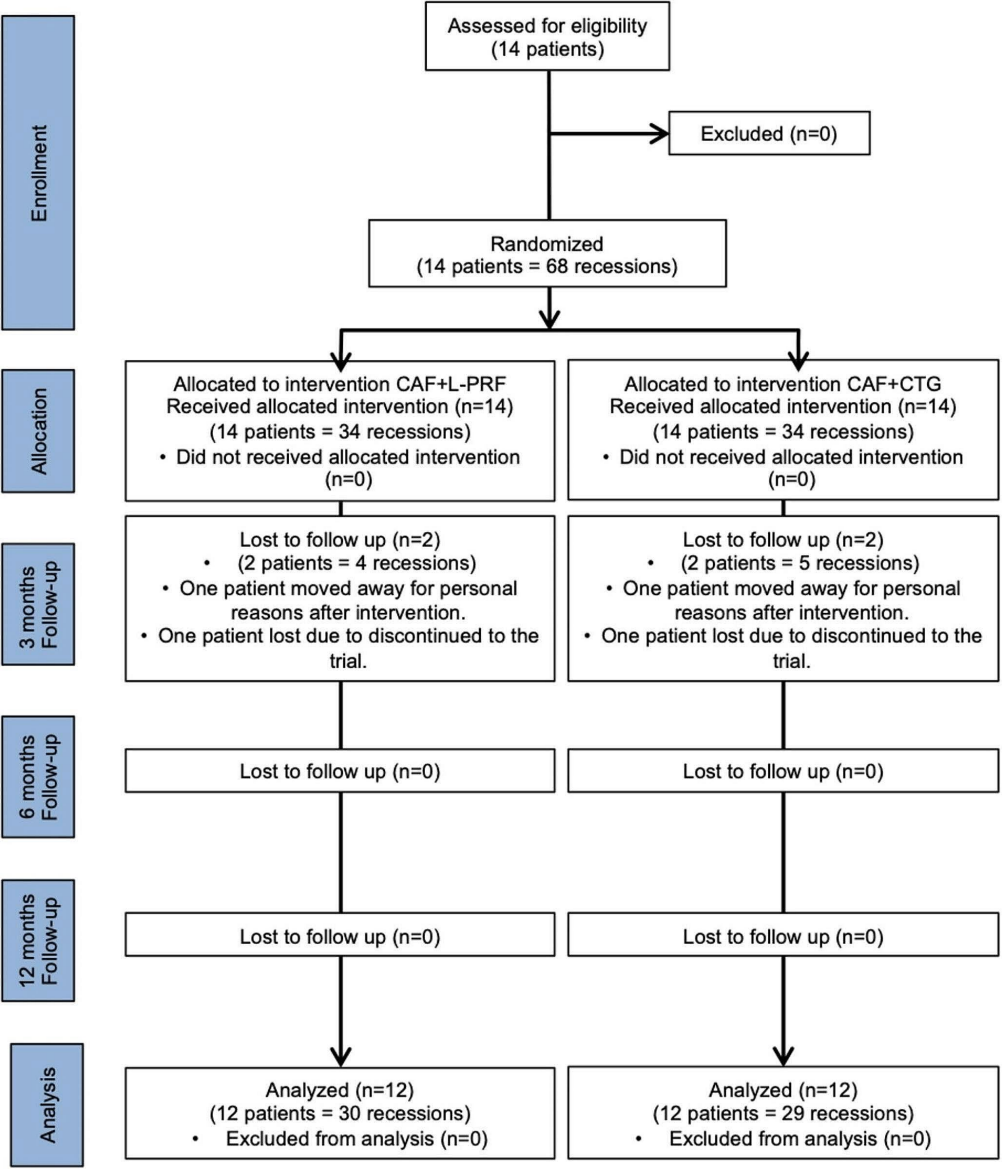
Flowchart of the study

The present study is a split-mouth, randomized, controlled, single blind clinical trial regarding the treatment of MAGRs. Two treatment groups; CAF + CTG (control) and CAF + L-PRF (test) were compared during a 12-month period. Figure 1 illustrates the flow chart of the current study. The Clinical Research Ethics Committee of Yeditepe University approved the study protocol on 22.04.2014 with the number 418. Before participating, all patients were provided with a comprehensive explanation of the purpose and procedures, and those who expressed a desire to participate provided written informed consent, according to Declaration of Helsinki from 1975, revised in 2013. On June 29, 2021, the study was officially registered on ClinicalTrials.gov with the identification number NCT04942821.

### Study population

A total of 14 patients with complaints of GRs were recruited from Marmara University Department of Periodontology between 2014 and 2015. According to the following eligibility criteria, subjects were enrolled to the study; having bilateral or contralateral two to four adjacent teeth with Miller Class I GRs [24] (Cairo classification RT1 [25]) ≥ 2 mm RD on non-molar teeth in the lower and / or upper jaws, being > 18 years old, periodontally healthy with full-mouth bleeding and plaque score ≤ 10% and no pocket depth (PD) > 3 mm, detectable cemento-enamel junction (CEJ), no previous periodontal surgery, systemically healthy with no situation of influencing coagulation or no use of any anticoagulant medicines, no blood-borne diseases, non-smokers with no history of smoking, no pregnancy, no lactation, not using antibiotics in the last 6 months, no presence of recession defects associated with caries, or pulpal pathology, deep abrasion, and restoration.

### Sample size

A statistical power analysis was conducted using a software program (Instat, Graphpad Software, USA). When the control and test groups were selected as dependents and the variables were assumed normally distributed, 22 recession defects were required in each group with a power of 80% at α error of 0.05 [26]. For potential dropouts, 34 recession defects were initially recruited per group.

### Pre-surgical intervention

After the screening, each participants received prophylaxis session, full mouth scaling and professional tooth cleaning. Recession defects were not to be surgically treated until patient could achieved an adequate level of plaque control. The patients were informed with non-traumatic “roll” technique using a soft manual toothbrush.

### Clinical measurements

A calibrated masked examiner (HSG) performed all measurements using periodontal probe (University of North Carolina, Hu-Friedy, Chicago, Illinois, USA). The treatment assignment and surgeries were not known by this examiner. Intra-examiner calibration was carried out in 10 patients with GR who were not included in the study. RD was measured twice, with a 1-day interval. Intra-examiner reliability was calculated as 0.94 for RD.

The following clinical measurements were performed at baseline and 3, 6, and 12 months after surgery.

- Plaque index (PI) [27] and gingival index (GI) [28].
- PD: at the mid buccal point of recession from the gingival margin (GM) to the bottom of the gingival sulcus.
- Clinical attachment level (CAL): at mid buccal point of the recession from CEJ to the bottom of the gingival sulcus.
- RD: at the mid buccal point of the recession from CEJ to GM.
- Recession width (RW): baseline measurement of the horizontal distance between the GM of the recession at CEJ level.
- Papilla width (PW): baseline measurement of the horizontal distance between the line angle of the two adjacent (mesial and distal) teeth at CEJ level.
- Gingival thickness (GT): at mid buccal point of the recession 3 mm apically from GM using #20 endodontic spreaders with silicone stoppers driven perpendicular to the tissue under topical anesthesia. The distance between silicon stopper and tip was measured with a digital calliper.
- KTW: at mid buccal point of the recession from free GM to mucogingival junction.

The following measurements were also recorded. Custom acrylic guides were used to provide a stable reference for these measurements.

- Position of CEJ (PCEJ): distance from the acrylic guide border to CEJ level before surgery.
- Position of GM (PGM): distance from the acrylic guide border to GM immediately after the surgery.

These measurements were used to calculate postoperative location of GM according to CEJ (GMCEJ = PCEJ – PGM).

Measurements were rounded up to nearest mm, out of GT which was recorded with endodontic spreader, calliper, and optical magnifier, at sensitivity of 0.1 mm.

Percentage of RC and CRC were performed with the following formulas.

Percentage of RC: [(Preoperative RD-Postoperative RD)/ Preoperative RD] x 100.

Percentage of CRC: [Teeth with CRC/all treated teeth] x 100.

Primary outcome of the study is percentage of RC. CRC, KTW and GT gains were the secondary outcomes of the study.

### Surgical procedure

All surgeries were performed by a periodontist (AY). Due to the design and nature of the study the periodontist and the participants were not blind to the surgical procedures. The MAGRs defects were randomly assigned to each site with toss of a coin on the operation day before the surgery. The recessions not included to the study were treated after completion of the study.

**Fig. 2:**
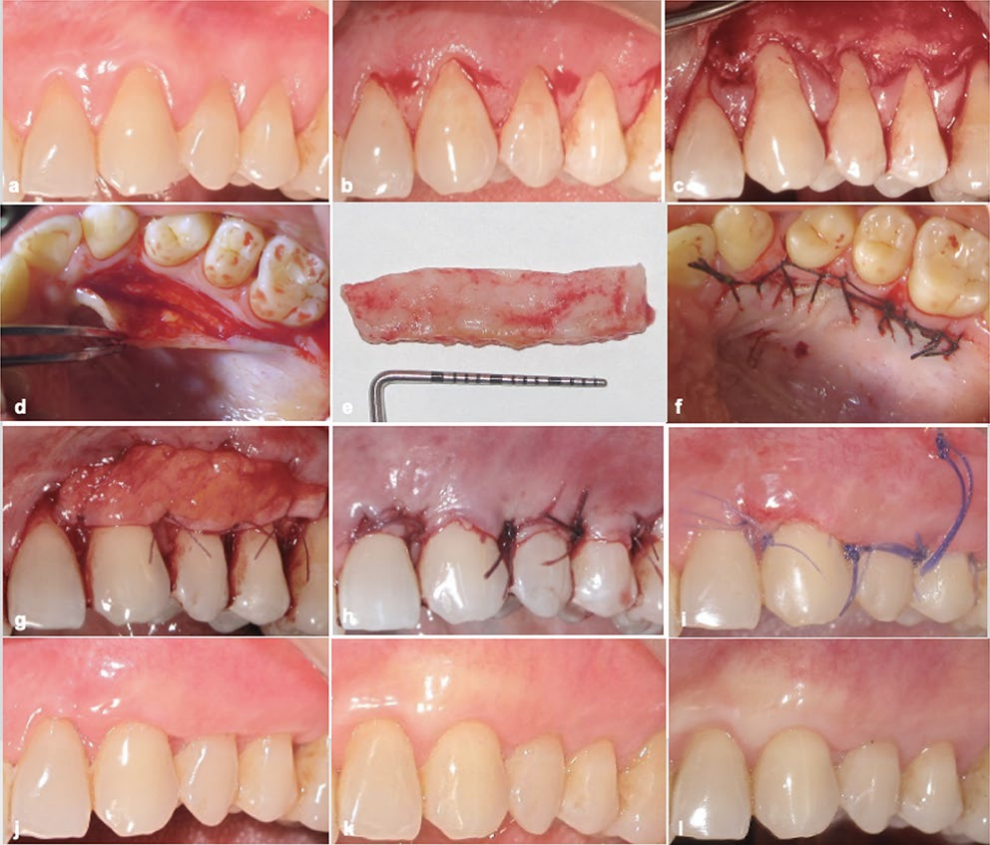
Coronally advanced flap + connective tissue graft site; **a** preoperative view, **b** incisions, **c** after flap elevation, **d-f** connective tissue graft harvesting, **g** after connective tissue graft suturing, **h** after flap suturing, **i** 2 weeks after surgery, **j** 3 months after surgery, **k** 6 months after surgery, **l** 12 months after surgery

**Fig. 3:**
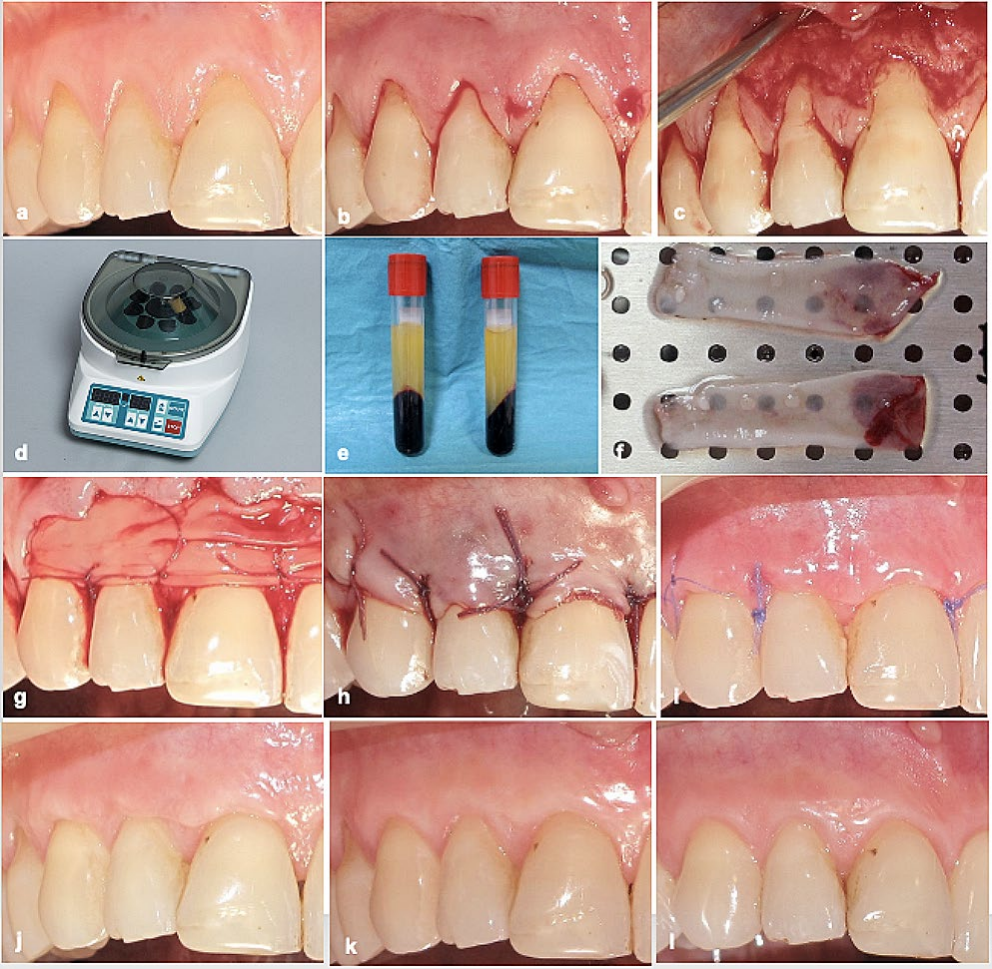
Coronally advanced flap + leucocyte- and platelet-rich fibrin site; **a** preoperative view, **b** incisions, **c** after flap elevation, **d-f** leucocyte- and platelet-rich fibrin preparation, **g** after leucocyte- and platelet-rich fibrin suturing, **h** after flap suturing, **i** 2 weeks after surgery, **j** 3 months after surgery, **k** 6 months after surgery, **l** 12 months after surgery

First CAF + CTG treatment as control, 6 weeks later CAF + L-PRF treatment as test were performed (Figs. 2a and 3a). A modified approach of CAF [29] was used in both treatment modalities (Figs. 2b-c and 3b-c). To provide a biocompatible surface for reattachment, root planing was applied to the exposed portion of the roots; the unexposed root surfaces were avoided in order to protect the periodontal attachment. CTG was harvested using single incision procedure as described by Hurzeler and Weng [30] (Fig. 2d-f). For L-PRF, blood was collected from the antecubital veins of patients using two 10 cc tubes with clot activator (BD Vacutainer CAT). Then, blood was centrifuged (Hettich EBA-20, Germany) at 2700 rpm for 12 min (Fig. 3d-e). PRF-BOX (Process, France) was utilized to form each fibrin clots into membrane (Fig. 3f). At the level of the CEJ, both CTG and two layers of L-PRFs were placed and sutured using 6/0 absorbable sutures (Pegelak, Doğsan, Türkiye) on the periosteum bed (Figs. 2g and 3g). Then, flap margins were positioned at least 0.5 mm coronally of the CEJ and sutured using 5/0 absorbable sutures (Pegelak, Doğsan, Türkiye) (Figs. 2h and 3h). Sutures were removed 2 weeks after each surgery (Figs. 2i and 3i).

### Post-operative care and follow-up

Patients were informed about postoperative care to protect surgical sites from infection and any trauma that may disrupt the wound stability and healing. Brushing was not performed on the sites for 2 weeks until suture removal, thereafter patients used ultra-soft post-operative brushes (Special Care, TePe, Sweden). Patients were prescribed 1000 gr amoxicillin with clavulanic acid (Augmentin BID, GlaxoSmithKline, Türkiye) every 12 h for 1 week and 0.2% chlorhexidine digluconate rinse (Klorheks, Drogsan, Türkiye) twice a day for 2 weeks. All patients were instructed to take oral analgesics (Etol Fort, Nobel İlaç, Türkiye) after the intervention every 8 h when necessary.

At the first month of post-surgical period, patients were recalled once a week and professional care was performed. Appointments were scheduled at the 3, 6 and 12 months in the follow-up period (Figs. 2j-l and 3j-l).

### Statistical analysis

All statistical tests were performed using SPSS software (IBM SPSS Statistics 20.0, IBM, NY, USA) at 95% confidence interval. Normality of the continuous variables was tested with Saphiro-Wilk test. For multiple intra-group observations, the ANOVA test was carried out if the data were normally distributed. Further paired t test was performed for paired comparison if significance was detected. Independent sample t test was used to compare continuous variables between groups. If the data did not have a normal distribution, the Friedman test was applied for multiple intragroup observations. Further Wilcoxon signed-rank test was performed for paired comparison if significance was detected. Mann Whitney U-test was used intergroup comparison between groups. Chi-square test was used to compare the distribution of operated teeth between groups. Since CRC is a nominal variable, chi-square test was used. The Spearman’s rank correlation analysis was applied to analyse the relation between the percentage of RC and CRC with study groups and recession defects’ specific parameters. Linear regression model was used to observe the factors affecting RC, logistic regression model was performed for CRC.

## Results

Out of 14 patients (68 recession defect), 2 patients (9 recession defects) were lost in the follow-up period. The study was completed with 12 patients (mean age 37.3 ± 9.42, 5 males and 7 females) and 59 recession defects (CAF + CTG group 29 and CAF + L-PRF group 30 recession defects). Healing was uneventful in both groups and no complications occurred after surgery. Table 1 shows the distribution of teeth type and location in the groups. No significant differences were observed between the groups regarding type of teeth involved and tooth location (*p* > 0.05). KTW were between 0.5 and 6 mm in all recession defects. Moreover, in CAF + L-PRF group 90% and in CAF + CTG 86% of the baseline RD were between 2 and 3 mm.

**Table 1:** Distribution of operated teeth according to groups

	Groups		*p*
	CAF + CTG *n* (%)	CAF + L-PRF *n* (%)	
*Type of teeth*			0.332
* Incisors*	3 (10.3)	7 (23.3)	
* Canine*	10 (34.5)	11 (36.7)	
* Premolar*	16 (55.2)	12 (40.0)	
*Tooth location*			0.797
* Maxilla*	15 (51.7)	14 (46.7)	
* Mandibulla*	14 (48.3)	16 (53.3)	
Total no. of tooth	29 (100.0)	30 (100.0)	

CAF: coronally advanced flap, CTG: connective tissue graft, L-PRF: leucocyte- and platelet-rich fibrin, Chi-square test, *p* < 0.05

Table 2 presents the clinical parameters of the operated sites at baseline and follow-up period. At baseline, no significant differences were observed in clinical parameters between both groups (*p* > 0.05). The comparison of PI and GI values within and between the groups at any follow-up time point revealed no statistically significant differences (*p* > 0.05). PD values were found to increase significantly in all time point in both groups (*p*<0.05). No differences were detected between the groups (*p* > 0.05). However, all the mean PD values were found to be within the healthy limits. CAL and RD values decreased significantly in both groups in all follow-up points after surgical treatment (*p*<0.05). Additionally, no significant difference was found between the groups at 3, 6 and 12 months after surgery (*p* > 0.05). Although the KTW value at 3 and 6 months in the CAF + CTG group was not significantly different from baseline, it increased significantly at 12 months (3.05 ± 0.77 mm) compared to baseline (2.62 ± 0.81 mm) (*p*<0.05). In the CAF + L-PRF group, KTW values decreased significantly from baseline (2.82 ± 1.27 mm) to the 3 and 6 months (2.30 ± 1.16 mm and 2.33 ± 1.18 mm, respectively) (*p*<0.05), on the other hand, 12-month value (2.75 ± 1.02 mm) was found to be similar with baseline (2.82 ± 1.27 mm) (*p* > 0.05). Significant increases in the KTW were detected in both groups at 12 months compared to their respective values at 3 and 6 months (*p*<0.05). The only significant difference in KTW between the CAF + CTG and CAF + L-PRF groups was found at 12 months (3.05 ± 0.77 mm and 2.75 ± 1.02 mm, respectively) (*p*<0.05). In the CAF + CTG group, GT increased significantly from the baseline to all postoperative follow-up periods (*p*<0.05). On the contrary, in the CAF + L-PRF group, no significant difference was found in GT between any follow-up period and baseline (*p* > 0.05). GT of the CAF + CTG group were significantly higher than the CAF + L-PRF group at 3, 6 and 12 months (*p*<0.05).

**Table 2:** Clinical parameters of treatment sites at baseline and follow-up visits

Variables	Time periods	Groups		*p*
		CAF + CTG *n* = 29 Mean ± SD	CAF + L-PRF *n* = 30 Mean ± SD	
PI^*a*^	Baseline	0.11 ± 0.18	0.11 ± 0.20	0.859
	3 months	0.15 ± 0.22	0.13 ± 0.22	0.655
	6 months	0.18 ± 0.28	0.14 ± 0.19	0.986
	12 months	0.18 ± 0.24	0.15 ± 0.24	0.477
	*p* ^*c*^	0.593	0.836	
GI^*a*^	Baseline	0.11 ± 0.20	0.03 ± 0.11	0.074
	3 months	0.18 ± 0.26	0.09 ± 0.21	0.065
	6 months	0.18 ± 0.27	0.09 ± 0.18	0.179
	12 months	0.16 ± 0.25	0.12 ± 0.23	0.439
	*p* ^*c*^	0.169	0.195	
PD (mm)^*a*^	Baseline	1.50 ± 0.57	1.33 ± 0.55	0.196
	3 months	1.91 ± 0.60^*^	2.08 ± 0.72^*^	0.395
	6 months	1.88 ± 0.46^*^	1.82 ± 0.55^*, #^	0.611
	12 months	1.83 ± 0.59^*^	1.60 ± 0.62^*, #^	0.147
	*p* ^*c*^	0.019	0.000	
CAL (mm)^*a*^	Baseline	4.09 ± 0.94	3.80 ± 0.75	0.161
	3 months	2.35 ± 0.77^*^	2.58 ± 1.01^*^	0.471
	6 months	2.28 ± 0.71^*^	2.38 ± 1.01^*^	0.918
	12 months	2.22 ± 0.86^*^	2.23 ± 0.83^*, #^	0.926
	*p* ^*c*^	0.000	0.000	
PW (mm)^*a*^	Baseline	3.79 ± 0.75	4.04 ± 0.10	0.62
RW (mm)^*a*^	Baseline	3.57 ± 0.80	3.35 ± 0.76	0.215
GMCEJ (mm)^*a*^	Surgery	1.09 ± 0.72	1.32 ± 0.89	0.76
RD (mm)^*a*^	Baseline	2.59 ± 0.84	2.47 ± 0.67	0.576
	3 months	0.43 ± 0.66^*^	0.53 ± 0.67^*^	0.504
	6 months	0.41 ± 0.52^*^	0.53 ± 0.69^*^	0.723
	12 months	0.40 ± 0.51^*^	0.63 ± 0.78^*^	0.326
	*p* ^*c*^	0.000	0.000	
KTW (mm)^*a*^	Baseline	2.62 ± 0.81	2.82 ± 1.27	0.692
	3 months	2.41 ± 0.54	2.30 ± 1.16^*^	0.127
	6 months	2.57 ± 0.78	2.33 ± 1.18^*^	0.138
	12 months	3.05 ± 0.77^*, #, †^	2.75 ± 1.02^#, †^	0.049
	*p* ^*c*^	0.000	0.000	
GT (mm)^*b*^	Baseline	1.11 ± 0.41	1.06 ± 0.32	0.625
	3 months	1.68 ± 0.35^*^	1.11 ± 0.28	< 0.001
	6 months	1.54 ± 0.31^*, #^	1.04 ± 0.27	< 0.001
	12 months	1.50 ± 0.33^*, #^	1.04 ± 0.28	< 0.001
	*p* ^*d*^	0.000	0.243	

CAF: coronally advanced flap, CTG: connective tissue graft, L-PRF: leucocyte- and platelet-rich fibrin, PI: plaque index, GI: gingival index, PD: probing depth, CAL: clinical attachment level, PW: papilla width, RW: recession width, GMCEJ: postoperative position of gingival margin according to CEJ, RD: recession depth, KTW: keratinized tissue width, GT: gingival thickness, n: number of tooth, SD: Standard deviation, ^a^Mann Whitney-U test, ^b^Independent sample t test, ^c^Freidman test, ^d^Repeated measures ANOVA test (with Bonferroni correction), ^*^Significant difference compared to baseline (Wilcoxon signed rank test-Paired t test), ^#^Significant difference compared to 3 month (Wilcoxon signed rank test-Paired t test), ^†^Significant difference compared to 6 month (Wilcoxon signed rank test- Paired t test), *p* < 0.05

Changes in PD, CAL, RD, KTW, GT, RC and CRC parameters are shown in Table 3. PD, RD reduction, and CAL gain of both groups were similar for all follow-up points (*p* > 0.05), in the favour of CAF + CTG group from baseline to 3-month, particularly. KTW gain from baseline to 12-month was significantly higher in the CAF + CTG group than the CAF + L-PRF group (*p*<0.05). After treatment, the CAF + CTG group consistently demonstrated significantly higher GT during all follow-up periods (*p* < 0.05). Notably, at the 12-month assessment, the CAF + CTG group exhibited a GT gain of 0.39 mm, whereas no gain was observed in the CAF + L-PRF group. Both groups had similar percentage of RC at all follow-up time periods (*p* > 0.05) with reaching out 83.7% and 76.9% at 12 months, respectively. CRC was achieved in 16 of 29 (55.2%) recessions treated with CAF + CTG, and 14 of 30 (46.7%) recessions treated with CAF + L-PRF at 12 months. However, no difference was observed between the two groups in CRC obtained at the end of the study (*p* > 0.05).

**Table 3:** Changes in clinical parameters after 3, 6 and 12 months

Variables	Time periods	Groups		*p*
		CAF + CTG *n* = 29 Mean ± SD	CAF + L-PRF *n* = 30 Mean ± SD	
PD reduction^*a*^	Δ0–3 month	-0.41 ± 0.77	-0.75 ± 0.77	0.127
(mm)	Δ0–6 month	-0.38 ± 0.69	-0.48 ± 0.58^*^	0.497
	Δ0–12 month	-0.33 ± 0.65	-0.27 ± 0.83^*^	0.78
CAL gain^*a*^ (mm)	Δ0–3 month	1.74 ± 0.85	1.22 ± 0.82	0.022
	Δ0–6 month	1.81 ± 1.00	1.42 ± 0.88	0.207
	Δ0–12 month	1.86 ± 0.93	1.57 ± 1.07	0.264
RD reduction^*a*^ (mm)	Δ0–3 month	2.15 ± 1.11	1.93 ± 0.65	0.317
	Δ0–6 month	2.17 ± 1.05	1.93 ± 0.58	0.411
	Δ0–12 month	2.19 ± 1.00	1.83 ± 0.60	0.228
KTW gain^*a*^ (mm)	Δ0–3 month	-0.20 ± 0.87	-0.51 ± 0.71	0.14
	Δ0–6 month	0.05 ± 1.10	-0.48 ± 0.74	0.083
	Δ0–12 month	0.43 ± 0.83^*^	-0.06 ± 0.85^*^	0.028
GT gain^*b*^ (mm)	Δ0–3 month	0.56 ± 0.36	0.04 ± 0.28	< 0.001
	Δ0–6 month	0.43 ± 0.35^*^	-0.02 ± 0.27^*^	< 0.001
	Δ0–12 month	0.39 ± 0.36^*^	-0.02 ± 0.20^*^	< 0.001
RC (%)^*a*^	3 months	81.8 ± 28.6	80.2 ± 23.9	0.607
	6 months	82.2 ± 22.6	80.8 ± 23.5	0.935
	12 months	83.7 ± 20.7	76.9 ± 26.3	0.383
	*p* ^*c*^	0.529	0.513	
CRC^*d*^ % (n)	3 months	62.1 (18)	53.3 (16)	0.601
	6 months	51.7 (15)	53.3 (16)	1
	12 months	55.2 (16)	46.7 (14)	0.606

CAF: coronally advanced flap, CTG: connective tissue graft, L-PRF: leucocyte- and platelet-rich fibrin, PD: probing depth, CAL: clinical attachment level, RD: recession depth, KTW: keratinized tissue width, GT: gingival thickness, RC: root coverage, CRC: complete root coverage, SD: Standard deviation, No: number, ^a^Mann Whitney-U test, ^b^Independent sample t test, ^c^Friedman test, ^d^Chi-square test, ^*^Significant difference compared to Δ0–3 month (Wilcoxon signed rank test-Paired t test), *p* < 0.05

RC (%) and CRC showed negative correlations with baseline RW, whereas positive correlation was observed with baseline PW and GMCEJ (*p* < 0.05) (Table 4). Moreover, regression analyses showed that both RC and CRC were positively associated with PW and GMCEJ, but negatively with RW (Tables 5 and 6) (*p*<0.05).

**Table 4:** Correlations between root coverage (%), complete root coverage at 12 months and clinical parameters at baseline

	RC (%)		CRC	
Parameters	*r*	*p*	*r*	*p*
Treatment groups	-0.144	0.277	-0.085	0.522
RD	-0.1	0.453	-0.143	0.281
RW	-0.3	0.021	-0.309	0.019
PW	0.354	0.006	0.345	0.007
KTW	-0.074	0.576	-0.054	0.684
GT	0.185	0.16	0.18	0.172
GMCEJ	0.348	0.007	0.336	0.009

RC (%): percentage of root coverage, CRC: complete root coverage, RD: recession depth at baseline, RW: recession width at baseline, PW: papilla width at baseline, KTW: keratinized tissue width at baseline, GT: gingival thickness at baseline, GMCEJ: postoperative position of gingival margin according to CEJ at baseline, correlation coefficient values by Spearman’s rank correlation test

**Table 5:** Linear regression analysis of the factors effecting root coverage at 12 months

Parameter	Estimate (B)	Std. Error.	Coefficient (t)	*p*
Intercept	67.932	15.394	4.413	0.000
PW	10.515	3.03	3.47	0.001
RW	-10.993	3.435	-3.2	0.002
GMCEJ	7.826	3.23	2.423	0.019

PW: papilla width at baseline, RW: recession width at baseline, GMCEJ: postoperative position of gingival margin according to CEJ at baseline

**Table 6:** Logistic regression analysis of the factors affecting complete root coverage at 12 months

Parameter	Beta	Std. Error	Odds Ratio	*p*
Intercept	-1.178	2.041	0.308	0.564
PW	1.044	0.463	2.839	0.024
RW	-1.141	0.545	0.319	0.036
GMCEJ	0.924	0.42	2.521	0.028

PW: papilla width at baseline, RW: recession width at baseline, GMCEJ: postoperative position of gingival margin according to CEJ at baseline

## Discussion

Achievement of predictable and aesthetic RC is the primary goal of periodontal plastic surgery. Although CTG is considered as a gold standard in RC procedure [2], it has some disadvantages including need for a second surgical area, postoperative bleeding, oedema, pain, flap necrosis at donor site and limited availability for the treatment of MAGRs [4, 5]. Recent years, the autologous alternative material, PRF, has become popular in the treatment of GR. In the present study, RC performance of L-PRF in MAGR was evaluated and compared with CTG in 12 months follow-up period.

Reducing interindividual variability between groups is crucial in treatment studies as it can potentially affect the baseline condition and/or treatment response. Thereby, split mouth study design was chosen in this study. Moreover, since the baseline characteristics of recession defects (RD, RW, KTW, and GT), as well as the distribution of tooth location and tooth type, were comparable in both treatment groups, the potential adverse impact of these defect-related factors on the study outcomes was reduced.

All patients showed successful maintenance of oral hygiene and periodontal health (PI ≤ 1, GI ≤ 1 and PD < 3 mm) during all follow-up periods. The results demonstrated that, both surgical techniques were effective in reducing RD thereby showing similar RC, CRC, CAL gain at 12-month. However, from baseline to 12 months higher GT and KTW gains were observed in the CAF + CTG group.

There are limited number of split-mouth studies about MAGR treatment with CTG and PRF at 12 month [20]. Most split-mouth studies have analysed either single GR or 6 months end point [14, 15, 18, 21–23]. The results of the previous studies suggest that the CAF + CTG is the gold standard technique for KTW gain [2, 31, 32]. CTG can potentially stimulate the development of keratinized tissue and maintain its original characteristics [33, 34]. In the present study, KTW in the CAF + CTG group remained stable from baseline to 6 months and increased about 0.5 mm at 12 months. However, in the CAF + L-PRF group, KTW decreased from baseline to 6 months but returned to its initial value at 12 months. While Özkan Şen and Öncü [23] reported that the KTW remained stable in both groups, Tunalı et al. [20] displayed an increase in both groups without any significant difference between them. In a recent meta-analysis, it has been reported that there is no additional significant positive benefit in CAF + PRF over CAF + CTG or CAF alone in providing KTW gain in multiple recession defects [35]. Moreover, it is important to note that our results indicate that KTW evaluation at 6-month may not accurately present the actual effect of the CTG or L-PRF. It is highly recommended to assess the impact of CTG or PRF on KTW at the 12-month mark, as the sole significant increase in KTW between groups was noted at this specific time point in our study.

GT has a positive impact on surgical outcomes, contributing to both RC and recession reduction. According to a previous study, CTG ensured better results compared to CAF alone, but this superiority was observed specifically when GT was less than 0.8 mm [36]. Furthermore, the beneficial effect of CTG usage was observed in the treatment of GR where a thin gingival phenotype exists [36]. However, study conducted by Stefanini et al. suggested using CTG selectively only for sites with GT < 1 mm and KTW < 1 mm [37]. This phenomenon may be attributed to a fundamental property of CTG, namely its role as a scaffold, facilitating the stabilization of blood clots and augmenting soft tissue thickness [32, 36]. In the present study, the baseline GT values were > 1 mm in both groups. Hence, our results exhibited a significant increase in GT in the CAF + CTG group at 3 months and persisted through the 12-month period whereas no change in the CAF + L-PRF group throughout the study. Previous studies disclosed a significant GT increase in both CAF + PRF and CAF + CTG groups 6-months after treatment of MAGRs [22, 23]. However, it should be kept in mind that in these studies either mean baseline GT was < 0.8 mm [22] or titanium-PRF (T-PRF) [23]was used. This suggests that the effectiveness of PRF in different gingival phenotypes is controversial or T-PRF and L-PRF may act differently in the alteration of the periodontal soft tissue phenotype. Another factor that might influence the GT gain is the thickness or the number of layers of the PRF used. However, a recent meta-analysis showed that there are no established guidelines regarding the optimal thickness of PRF for treatment of Miller class I and II (or Cairo RT 1) GRs or the number of PRF membranes required per site [38]. In our study, two layers of membrane were utilized, whereas other studies used only one layer [14, 19, 20, 22, 23]. Despite this, the PRF did not demonstrate any positive effect on GT gain. This may be attributed to the fact that the thickness of the L-PRF membrane layer may vary from person to person due to significant individual differences. Therefore, the impact of PRF thickness and/or layers on GT requires further clarification in future studies.

The key parameters in predictable and successful root coverage surgeries are CRC, RC, or RD reduction. In the present study, both surgical procedures resulted in significant RD reductions from baseline to 12 months (2.19 ± 1.00 mm and 1.83 ± 0.60 mm, respectively), and almost 80% of RC, 50% of CRC at 12 months without any difference between the groups. Available data indicate that the rate of RC obtained by the use of CTG and PRF in GR treatment ranges from 60 to 98% [15, 18, 20–23] and CRC ranges from 20 to 90% [15, 18, 20–22]. Additionally, some studies reported no significant difference in terms of the RD reduction, RC and CRC between CTG and PRF procedures in patients with single GR [15, 18] or MAGRs [20–22]. Although the study designs were similar, the difference in the number of patients, baseline defect characteristics or the baseline RD might affect the RC and CRC outcomes. Based on the outcomes regarding the RD reduction, RC and CRC in the current study, the CAF + L-PRF technique demonstrated comparable success to the CAF + CTG technique at the 12-month. This finding suggests that L-PRF could serve as an alternative to CTG in the treatment of MAGRs.

The effect of anatomical, patient-related, and technic-related factors on the clinical outcome of surgical procedures have been investigated in several studies [39]. Limited to 2 to 3 mm Miller I MAGRs our findings indicated that CTG or L-PRF application have no association with RC and CRC. However, regardless of surgical technique, initial PW, RW, and GMCEJ were detected to be crucial parameters affecting the RC and CRC outcomes in the treatment of GR. These findings are in consistent with numerous previous studies [40–42]. Therefore, initial PW, initial RW, and GMCEJ should be taken into consideration to predict the percentage of RC and CRC 12 months after treatment.

The strength of the current study is the extended recovery periods of GR patients, reaching up to 12 months in a split-mouth design. However, limitations include the lack of postoperative patient-centered outcome and aesthetic evaluation by both clinicians and patients. Moreover, in most of the included tooth sites the RD was limited to between 2 and 3 mm.

## Conclusion

Within the limits of present study, both CAF + L-PRF and CAF + CTG procedures are effective techniques in the treatment of MAGRs in terms of RC, CRC, and CAL gain after 12 months. The L-PRF can be a promising alternative to the CTG in the treatment of MAGRs. Further randomized, controlled clinical studies including MAGRs sites with > 3 mm RD need to be performed to confirm our findings.

## Data Availability

The data that support the findings of this study are available from the corresponding author upon reasonable request.
